# Safety Comparison of Risk of Liver Dysfunction between Generic and Brand Statin Drugs Marketed in Japan: A Cohort Study Using MID-NET®

**DOI:** 10.1007/s43441-025-00904-w

**Published:** 2025-12-27

**Authors:** Hotaka Maruyama, Yuki Kinoshita, Takashi Ando, Jun Okui, Maki Komamine, Kazuhiro Kajiyama, Naoya Horiuchi, Yoshiaki Uyama

**Affiliations:** 1 Office of Pharmacovigilance I, Pharmaceuticals and Medical Devices Agency, Tokyo, Japan; 2 Office of Regulatory Science Research, Center for Regulatory Science, Pharmaceuticals and Medical Devices Agency, Tokyo, Japan; 3 Office of Medical Informatics and Epidemiology, Pharmaceuticals and Medical Devices Agency, Tokyo, Japan; 4 Present Address: Center for Regulatory Science, Pharmaceuticals and Medical Devices Agency, Tokyo, Japan

**Keywords:** Generic drugs, Statin, Liver dysfunction, Database Study, Pharmacoepidemiology, High dimensional propensity score

## Abstract

To verify safety profiles of generic statins in a real-world setting, the risk of liver dysfunction as a common adverse event was compared between generic and brand drugs. A new user cohort design was employed in which patients prescribed one of six statins (atorvastatin, simvastatin, pitavastatin, pravastatin, fluvastatin, or rosuvastatin) in Japan between January 1, 2014 and March 31, 2022 were identified in the MID-NET^®^ database. Adjusted hazard ratios (aHRs) for generic drugs compared with their corresponding brand drug for the occurrence of first liver dysfunction were estimated using high-dimensional propensity score-weighted Cox models. Among the six statins, no increased trends in aHRs were observed in the primary analysis, except for atorvastatin. The primary analysis showed an aHR of 2.08 (95% confidence interval [CI]: 1.20–3.63) for atorvastatin. In an additional analysis with shorter follow-up periods, aHRs for atorvastatin gradually approached 1.00 (1.74 [95% CI: 0.94–3.22] within 360 days, 1.65 [95% CI: 0.84–3.25] within 180 days, 1.49 [95% CI: 0.73–3.01] within 90 days, and 1.33 [95% CI: 0.60–2.96] within 30 days). Results suggest that risks of liver dysfunction by generic statins are similar to those for brand drugs, facilitating our understanding about the safety of generic drugs. The aHR for atorvastatin was inconsistent between the primary and additional analyses, which suggests that the observed increased risk of generic atorvastatin may be affected by other factors and does not necessarily indicate a different safety profile between generic and brand drugs.

**Supplementary Information:**

The online version contains supplementary material available at 10.1007/s43441-025-00904-w.

## Introduction

Use of generic drugs in Japan has recently increased following a government initiative on the basis of equivalent efficacy and safety between a generic drug and its corresponding brand drug [1–3]. The safety profile of a generic drug is generally considered as well-established at the time of approval because of accumulated safety information for the corresponding brand drug in clinical trials and post-marketing studies. However, serious cases of adverse reaction due to contamination in the manufacturing process of a generic drug have been recently reported [4] and the latest government survey in Japan indicated that some of patients have a distrust and concerns about using generic drugs because of a preconception that generic drugs are inferior or different to brand drugs in terms of quality, efficacy, and safety [5].

To overcome patients’ distrust and concerns, and facilitate a proper understanding of generic drugs, more scientific evidence should be presented to the public in Japan. Many studies on the clinical effectiveness and safety of generic drugs compared to brand drugs have been reported in the US and other countries [6–14], but few have been reported in Japan [15, 16]. Therefore, to provide further scientific evidence on the safety of generic drugs in Japan, we conducted an observational database study comparing the safety of generic drugs with that of relevant brand drugs by using real-world data (RWD) from MID-NET^®^.

For this study, statins were selected as target drugs because a sufficient number of patients, prescribed generic and brand drugs on a daily basis over a long period, was necessary to appropriately compare the risks between generic and brand drugs. During the planning stage of this study, we confirmed that statins met these conditions based on available information in Japan, such as patient surveys and the survey of medical care expenditure [17, 18]. Similarly, for the outcome, a sufficient number of events was necessary for the analysis. Liver dysfunction was considered appropriate because it is a common adverse drug reaction to statins, occurring in more than a few percent of Japanese patients, as described in Japanese package insert for statins[19, 20]. Moreover, the risks of liver dysfunction could be objectively evaluated using laboratory test results available in MID-NET^®^ [21].

## Materials and Methods

### Database

In this study, RWD from MID-NET^®^, a reliable and valuable database in Japan, were used for the analysis because MID-NET^®^ stores electronic medical records, administrative claim data, and diagnosis procedure combination data of more than 5.7 million patients (as of December 2021) in cooperation with 10 healthcare organizations, including 23 university hospitals and regional core hospitals [21, 22]. In this database, data on laboratory tests of liver function, which are useful for detecting liver dysfunction, were available for analysis. The study period spanned from January 1, 2013 to March 31, 2022.

Utilization of MID-NET^®^ for this study was approved on July 1, 2022 through a discussion by the expert committee of MID-NET^®^ [23]. The actual data extraction from MID-NET^®^ for analysis was carried out between August 23, 2022 and August 31, 2022. The anonymized dataset was provided only to the authors from the MID-NET^®^ management office. Since this study was conducted as an official activity of the PMDA under the Pharmaceuticals and Medical Devices Agency Law [Article 15–5–(c) and (f)]c, it was not subject to review by institutional review boards in accordance with the Japanese ethical guidelines. Written informed consent from the participants’ legal guardian/next of kin was not required in this study in accordance with national legislation and institutional requirements [24, 25].

### Study Population

We employed a new user cohort design. This study included patients who met all of the following criteria: (i) patients newly prescribed at least one of six statins (atorvastatin, simvastatin, pitavastatin, pravastatin, fluvastatin, or rosuvastatin) during the period from January 1, 2014 to March 31, 2022, (ii) patients with any medical records at least 181 days or more before t_0_ (the earliest prescription date of the statins), and (iii) patients with the diagnosis of dyslipidemia at or before t_0_.

Patients who met one or more of the following criteria were excluded from the analysis: (i) patients prescribed multiple statins at t_0_, (ii) patients without information about the brand or generic name of the statins at t_0_, (iii) patients prescribed any anticancer drugs or with a record of radiotherapy during the look-back period (i.e., 180-day period on or before t_0_), (iv) patients prescribed any antiviral drugs for hepatitis B or C during the look-back period, (v) patients with grade 2 or higher liver dysfunction (same definition as the primary outcome) (see “Outcome Definition and Follow-up Period”) during the look-back period, and (vi) patients only with a record at t_0_ (0 follow-up days) (see Supplementary Figure S1 for more details of the study design).

Patients were classified into six cohorts based on the statin prescribed at t_0_. The generic and brand groups were defined as patients prescribed generic and corresponding brand statins, respectively. It should be noted that among the generic drugs for pitavastatin and rosuvastatin, there were some drugs whose active ingredients, excipients, manufacturing processes, and manufacturing sites were identical to those of the corresponding brand drugs [26, 27]. These generic drugs were classified as brand drugs. For rosuvastatin, comparisons between generic and brand drugs were mainly conducted using data on and after December 8, 2017 as generic rosuvastatin was launched in Japan on that date. All patients prescribed rosuvastatin before December 8, 2017 were classified as a historical brand group for secondary analysis.

### Outcome Definition and Follow-up Period

This study focused on the risk of liver dysfunction because it was one of the common adverse events associated with statins. The primary outcome of liver dysfunction was defined according to the Common Terminology Criteria for Adverse Events (CTCAE) v5.0 [28, 29] as the first occurrence date during the follow-up period of (i) grade 2 or higher aspartate aminotransferase (AST) and alanine aminotransferase (ALT) on the same day, or (ii) grade 2 or higher total bilirubin (T-BIL), alkaline phosphatase (ALP), and gamma-glutamyl transferase (GGT) on the same day. For the secondary outcome, target severity of the outcome was changed from grade 2 to grade 3.

The follow-up period started one day after t_0_ and ended at the earliest date among the following: (i) 90 days after the last prescription end date, (ii) the day before the date of a different statin prescription including a switch to brand or generic drug with the same active ingredient, (iii) the date of the last medical record for a patient during the study period, or (iv) the date of the first occurrence of the outcome. The last prescription end date was defined using the gap period between the end date of the preceding prescription and the start date of the subsequent prescription. If the gap period was within 90 days, it was considered a continuous prescription period for the patient. The reason we chose 90 days as the gap period was that the longest period per prescription is limited to 90 days in Japan.

### Covariates

Covariates were selected by the high-dimensional propensity score (HDPS) method [30] in addition to basic 18 covariates for each cohort, and each basic covariate was dichotomized (see Table 1). Basic covariates included: sex and age (65 years or older) at t_0_; history of liver dysfunction 1 (AST and ALT); history of liver dysfunction 2 (T-BIL, ALP, and GGT); history of renal dysfunction (estimated glomerular filtration rate [eGFR] and creatine kinase [CK]); history of laboratory dysfunction related to dyslipidemia (low density lipoprotein cholesterol [LDL], high density lipoprotein cholesterol [HDL], and triglyceride [TG]); history of lipid-lowering drugs other than statins; history of complications related to dyslipidemia (hypertension, diabetes mellitus, occlusive arteriosclerosis, coronary artery disease, cerebrovascular disease, renal disease); and history of fatty liver disease during the look-back period.

**Table 1:** Characteristics of patients prescribed atorvastatin (primary analysis)

Variables, n (%)		Unadjusted					Adjusted				
		Generic (n = 12,821)		Brand (n = 2,957)		ASD^†^	Generic (n = 12,737)		Brand (n = 13,570)		ASD^†^
Sex											
Male		6,755	(52.7)	1,343	(45.4)	0.146	6,699	(52.6)	6,528	(48.1)	0.090
Age group (years)											
≧65		8,533	(66.6)	1,772	(59.9)	0.138	8,470	(66.5)	9,460	(69.7)	0.067
Laboratory test result category											
Liver functions1^†^	Grade1	2,883	(22.5)	608	(20.6)	0.047	2,856	(22.4)	3,453	(25.4)	0.074
Liver functions2^†^	Grade1	3,212	(25.1)	775	(26.2)	0.026	3,186	(25.0)	3,275	(24.1)	0.020
eGFR^†^	< 60 mL/min/1.73m^2^	5,375	(41.9)	1,184	(40.0)	0.038	5,329	(41.8)	6,467	(47.7)	0.118
Creatinine Kinase	≧ULN^†,‡^	1,411	(11.0)	268	(9.1)	0.065	1,391	(10.9)	1,898	(14.0)	0.102
Low Density Lipoprotein	≧140 mg/dL	1,510	(11.8)	253	(8.6)	0.107	1,487	(11.7)	1,405	(10.4)	0.044
High Density Lipoprotein	< 40 mg/dL	2,922	(22.8)	737	(24.9)	0.050	2,910	(22.8)	2,746	(20.2)	0.061
Triglyceride	≧150 mg/dL	4,144	(32.3)	922	(31.2)	0.025	4,119	(32.3)	4,384	(32.3)	0.001
Medications for dyslipidemia											
Other than statins	Yes	1,148	(9.0)	279	(9.4)	0.017	1,139	(8.9)	1,048	(7.7)	0.042
Comorbidities											
Hypertension	Yes	8,266	(64.5)	1,746	(59.0)	0.112	8,194	(64.3)	8,404	(61.9)	0.049
Diabetes	Yes	8,539	(66.6)	2,014	(68.1)	0.032	8,478	(66.6)	8,789	(64.8)	0.038
ASO^†^	Yes	2,110	(16.5)	496	(16.8)	0.008	2,094	(16.4)	2,244	(16.5)	0.003
CAD^†^	Yes	3,448	(26.9)	639	(21.6)	0.124	3,407	(26.7)	3,350	(24.7)	0.048
CVD^†^	Yes	4,897	(38.2)	948	(32.1)	0.129	4,844	(38.0)	5,178	(38.2)	0.003
Renal disease	Yes	2,510	(19.6)	716	(24.2)	0.112	2,484	(19.5)	2,819	(20.8)	0.031
Fatty liver disease	Yes	484	(3.8)	123	(4.2)	0.020	482	(3.8)	488	(3.6)	0.010
Other liver disease	Yes	1,752	(13.7)	500	(16.9)	0.090	1,746	(13.7)	1,551	(11.4)	0.063

^†^ASD, absolute standardized means difference; ASO, arteriosclerosis obliterans; CAD, coronary artery disease; CVD, cerebral vascular disease; eGFR, estimated glomerular filtration rate; Liver functions 1, aspartate aminotransferase (AST) or alanine aminotransferase (ALT); Liver functions 2, gamma glutamyl transferase (GGT), total-bilirubin (T-Bil) or alkaline phosphatase (ALP); ULN, upper limit normal

^‡^ULN, 248 U/L (male), 153 U/L (female)

For the HDPS method, three dimensions assessing medication, disease and medical procedure were defined based on information in the look-back period. The coding systems used for each dimension were the first five digits of the ATC code [31] for medications, the first three digits of the international statistical classification of diseases and related health problems 10th revision (ICD-10 code) [32] for diseases, and the first four digits of the Japanese Procedure Code (including administrative fee, surgery and procedure, etc.) for medical procedures. Candidate covariates were prioritized using an indicator variable based on code occurrences, and the selection was restricted to the 200 most prevalent codes in each dimension. To satisfy the positivity assumption, covariates with zero patients in either the generic or brand group were excluded. Finally, covariates including basic 18 covariates and covariates identified through the HDPS method amounting to one-tenth of the lower number of patients in either generic or brand group were selected using Bross’s formula with zero-cell correction.

### Statistical Analysis

The patient characteristics were summarized for basic covariates, and differences between groups were assessed by calculating the absolute standardized mean difference (ASD) [33]. The median follow-up period with an interquartile range (IQR) was tabulated for each group. Regarding the occurrence of liver dysfunction, the number of patients with the outcome during the follow-up period and the median duration to the occurrence of outcome with IQR was also tabulated for each group.

To compare the risk of liver dysfunction between generic and brand groups in each cohort, a Cox proportional hazards model was used to estimate crude and adjusted hazard ratios (aHR) and 95% confidence intervals (95% CI). Multivariable logistic regression model was used to calculate the HDPS, and standardized mortality ratio weight based on the HDPS was applied for estimating aHR in the population limited to the overlapped area of the HDPS between the generic and the brand groups. It should be noted that in the fluvastatin cohort, all analyses were performed using propensity scores calculated only with basic 18 covariates rather than HDPS due to the small sample size.

In the secondary analysis, for examining effects of exposure misclassification, the crude and aHRs were estimated by comparing patients prescribed generic rosuvastatin with the historical brand group (brand rosuvastatin) (see “Study Population”), because this study used prescription data but not dispensing data, and actual drug taken by a patient may have been changed from brand to generic drug or generic to brand drug at the pharmacy. In this analysis, instrumental variables defined as the cut-off value of log (RR_CE_: risk ratio for the covariate-exposure) > 1.5 and log (RR_CD_: risk ratio for the covariate-outcome) < 0.5 [34, 35] were excluded for aHR calculation to minimize bias because a large difference in HDPS distribution was only observed in the comparison with the historical control group, probably due to time-dependent factors such as newly approved drugs.

Moreover, an additional analysis was conducted to examine the possibility that the primary analysis may have included a late-onset outcome unrelated to statins. This is because latency of drug induced liver injury (DILI) has been typically reported as a range between 5 days and 3 months after starting statins and differences in the time to event (liver dysfunction) were observed between generic and brand groups, especially in the atorvastatin cohort (see “Results” and Table 2). In the additional analysis, another criterion was added to end of the follow-up period, which was censored on the dates of 30, 90, 180, or 360 days from t_0_.

SAS Version 9.4 (SAS Institutes, Cary, NC, USA) was used for all analyses.

**Table 2:** Summary statistics in each cohort about number of events, follow-up period and the time to event on primary outcome in the adjusted population

Cohort*	Group	Number of events		Follow-up period (days)		Time to event (days)	
		n^‡^	(%^‡^)	median	(IQR^†^)	median	(IQR^†^)
ATOR	Generic	398	(3.12)	145	(98–479)	57	(13–304)
	Brand	145	(1.07)	99	(16–215)	15	(10–48)
SIMV	Generic	< 10	(^‡^)	92	(9–105)	7	(3–8)
	Brand	< 10	(^‡^)	98	(46–123)	46	(7–58)
PITA	Generic	160	(3.26)	138	(96–422)	54	(9–265)
	Brand	157	(2.97)	119	(63–307)	7	(3–67)
PRAV	Generic	105	(2.78)	123	(94–365)	53	(8–493)
	Brand	83	(1.92)	100	(11–167)	115	(9–535)
FLUV	Generic	< 10	(^‡^)	98	(32–157)	157	(157–157)
	Brand	< 10	(^‡^)	119	(95–427)	140	(73–563)
ROSV	Generic	44	(1.99)	117	(52–294)	26	(8–147)
	Brand	48	(2.16)	125	(93–329)	48	(7–125)

*ATOR, atorvastatin; SIMV, simvastatin; PITA, pitavastatin; PRAV, pravastatin; FLUV, fluvastatin; ROSV, rosuvastatin, ^†^IQR, Interquartile range

^‡^Data are masked so that the number of patients (less than 10) cannot be identified according to the MID-NET^®^ publication criteria

## Results

### Study Population and Patient Characteristics

**Fig. 1:**
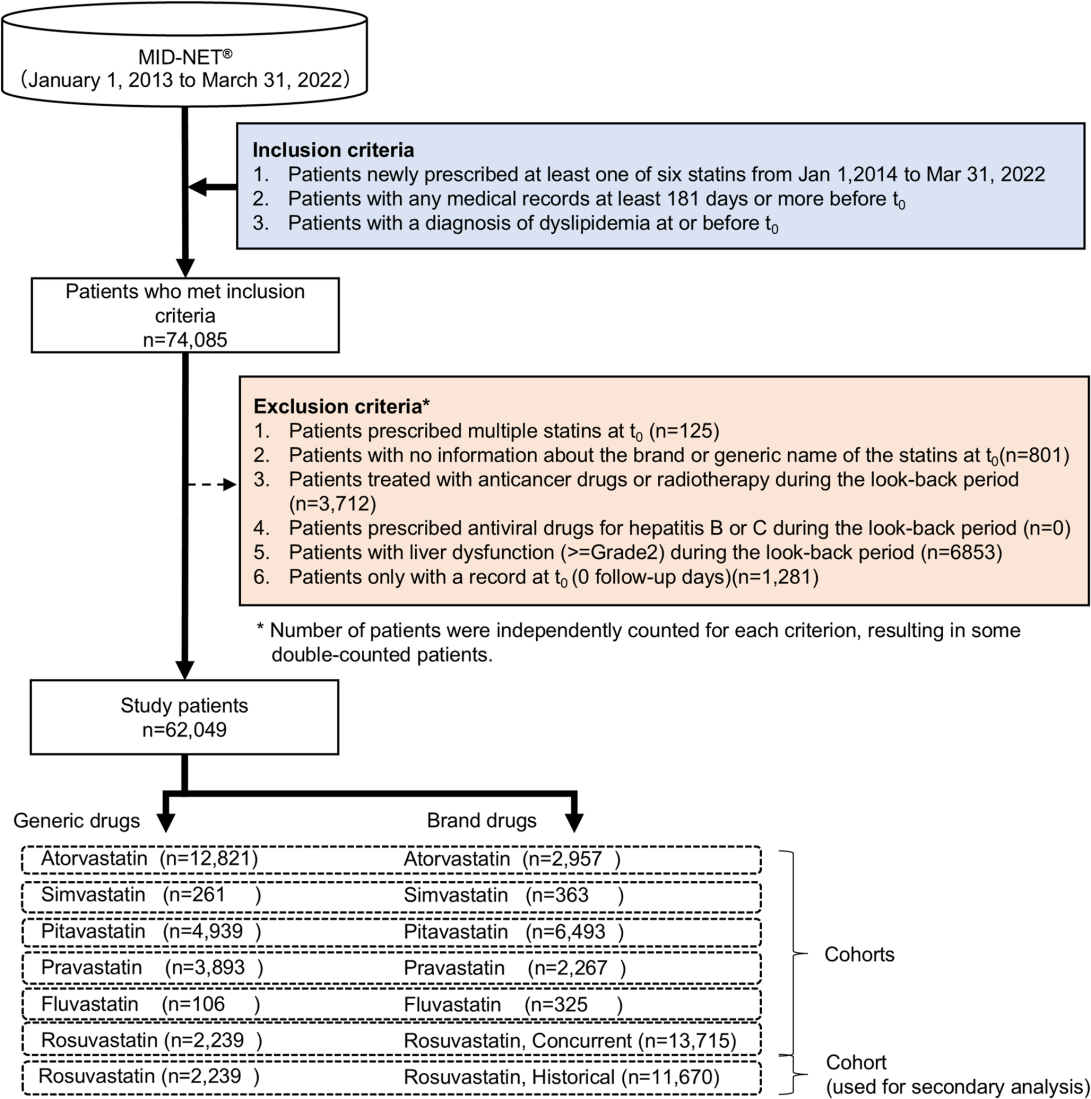
Study flowchart for patient selection

As shown in Fig. 1, of 74,085 patients who met inclusion criteria, 62,049 patients were included for the analysis after the exclusion criteria were applied. The rosuvastatin cohort had the highest number of patients (generic group: n = 2,239, brand group as concurrent control: n = 13,715), followed by the atorvastatin, pitavastatin, and pravastatin cohorts. It is noteworthy that the number of patients prescribed a generic statin drug was the highest for atorvastatin (n = 12,821), and the numbers of patients prescribed simvastatin or fluvastatin were relatively small.

Patient characteristics before and after adjustment for basic covariates in the atorvastatin cohort, which had the largest number of patients prescribed the generic drug, are shown in Table 1. In the adjusted population, patient backgrounds were generally well-balanced (ASD < 0.1), except for the proportion of patients with renal dysfunction (i.e., eGFR and CK), which was slightly lower in the generic group than in the brand group. Similarly, for other statins, no major differences in patient backgrounds were observed between the generic and brand groups, except for the proportion of patients with hypo-HDL cholesterolemia, which was slightly higher in the generic group than in the brand group in the pravastatin cohort, although the ASD for this case was below 0.2 (see Supplementary Tables S1.1–S1.5 for patient characteristics of other statins).

**3.2. Summary Statistics (Number of Events, Follow-up Period and Time to Even**t).

Summary statistics on number of events, follow-up period, and time to event are shown in Table 2. In the adjusted population, the median follow-up period in each cohort was in the range of 92–145 days with large variability in IQR, but indicated no major differences between generic and corresponding brand groups. In the case of atorvastatin, the median follow-up period was 145 days in the generic group and 99 days in the brand group, which was relatively a large difference between the groups. Median time to event (liver dysfunction) in each cohort was in the range of 7–157 days with large variability in IQR. For example, the median time to event (IQR) was 57 (13–304) days in the generic group and 15 (10–48) days in the brand group for the atorvastatin cohort, 54 (9–265) days in the generic group and 7 (3–67) days in the brand group for the pitavastatin cohort, and 53 (8–493) days in the generic group and 115 (9–535) days in the brand group for the pravastatin cohort.

### Risk of Liver Dysfunction

**Fig. 2:**
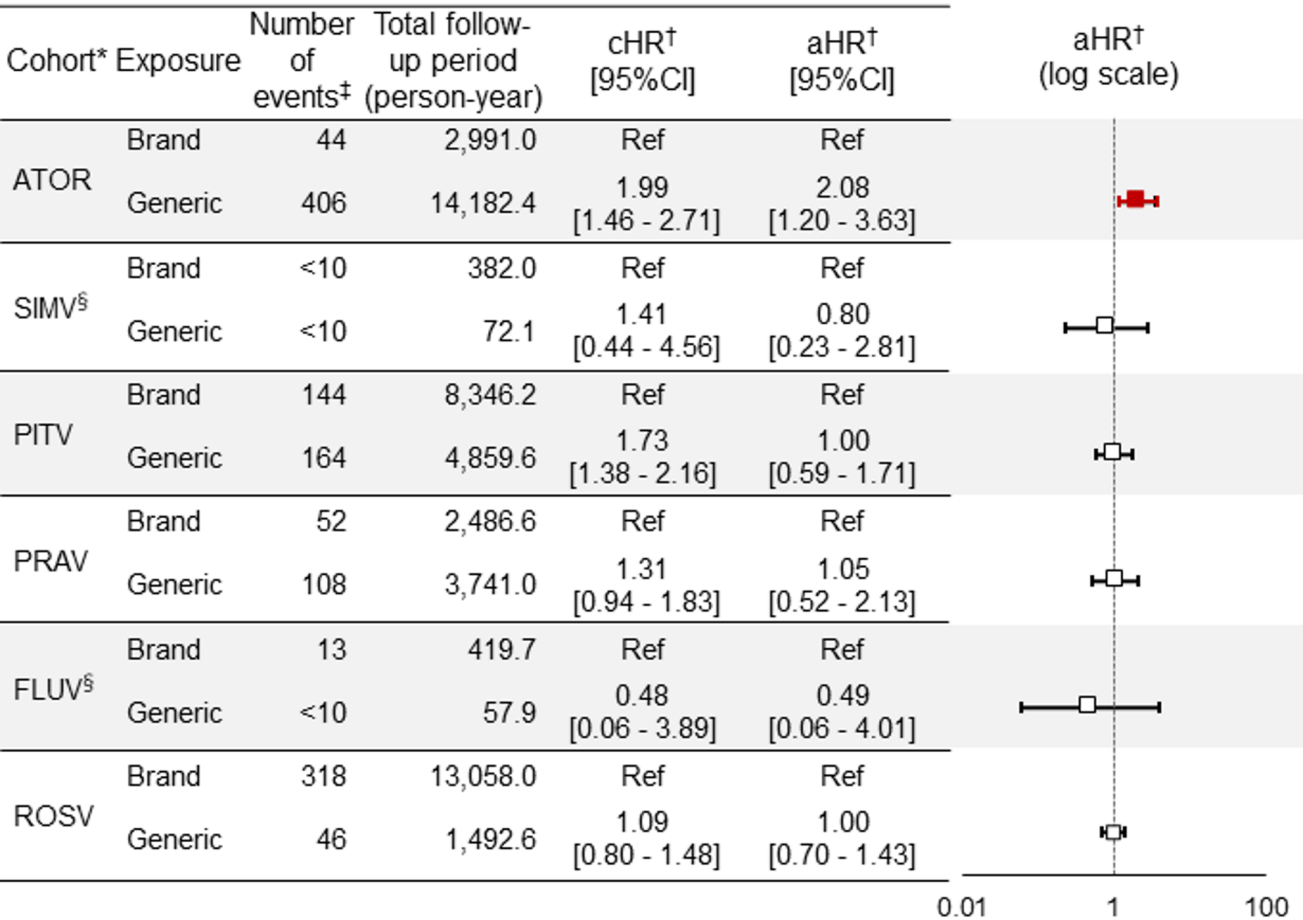
Comparisons of risk of liver dysfunction between generic and brand drugs of various statins on the primary outcome. *ATOR: atorvastatin, SIMV: simvastatin, PITA: pitavastatin, PRAV: pravastatin, FLUV: fluvastatin, ROSV: rosuvastatin. † cHR: crude hazard ratio, aHR: adjusted hazard ratio, Ref: reference. ‡ Data are masked so that the number of patients (less than 10) cannot be identified according to the MID-NET^®^ publication criteria. § The estimation accuracy of the adjusted hazard ratio was low for the group due to the small number of patients

The risk of liver dysfunction, the primary outcome, was compared between generic and brand groups of various statins (Fig. 2). Higher aHR with 95% CI exceeding 1.00 for the generic group compared with the brand group was observed only in the atorvastatin cohort (aHR: 2.08, 95% CI: 1.20–3.63), and aHRs in the other five cohorts ranged from 0.49 to 1.05 with their 95% CIs including 1.00. The results for rosuvastatin (aHR: 1.00, 95% CI: 0.70—1.43) were also consistent with results in the secondary analysis (comparison with the historical brand group, aHR: 0.96, 95% CI: 0.65—1.42).

Results for the secondary outcome were similar to those for the primary outcome in which aHR was higher only in the atorvastatin cohort (aHR: 2.54, 95% CI: 1.35—4.75), although the 95% CI was wider for the simvastatin and pravastatin cohorts due to the small number of events. The aHR could not be calculated for the fluvastatin cohort because no outcome was observed in the generic group (see Supplementary Figure S2 for more details).

**Fig. 3:**
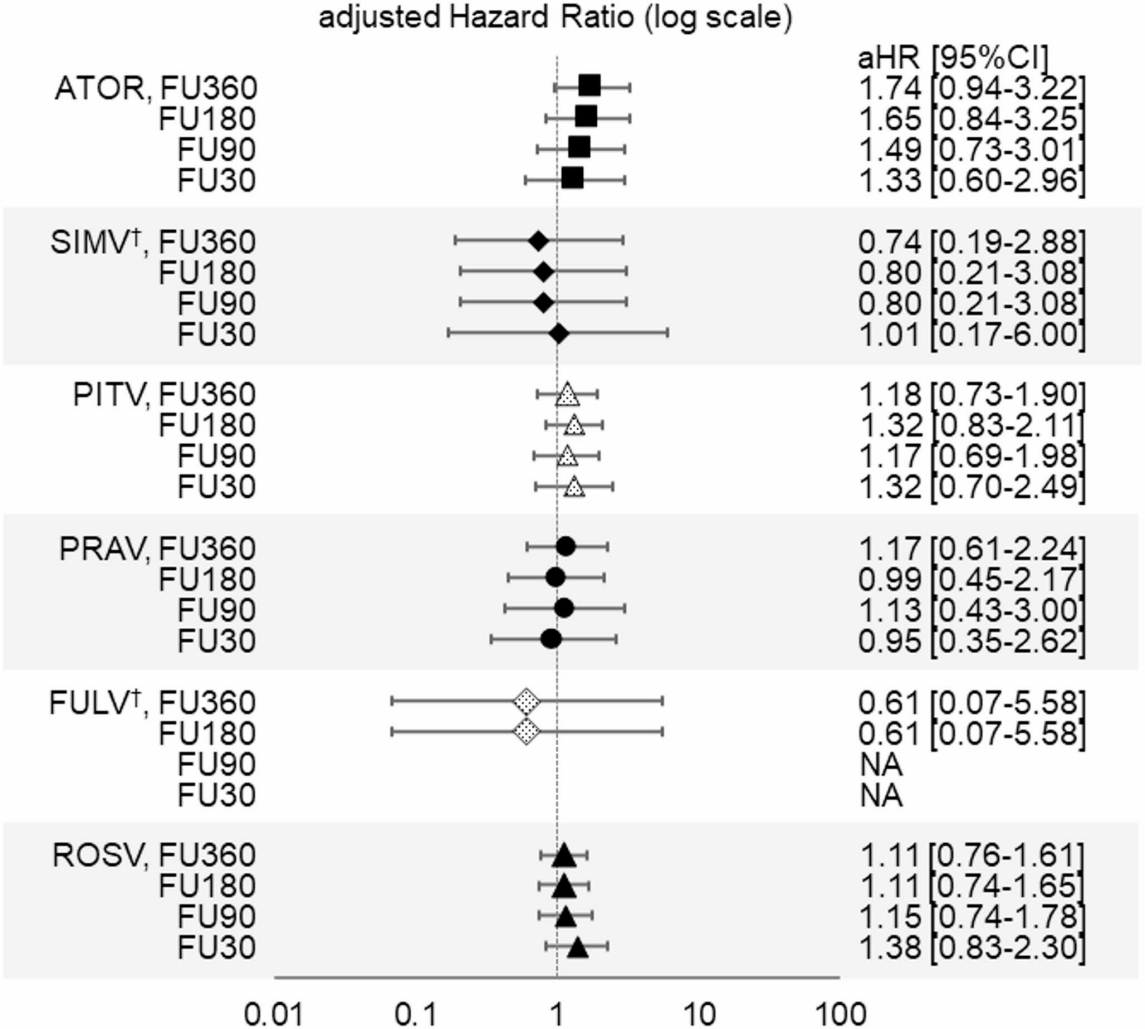
aHRs on the risk of liver dysfunction by various statins in comparison between generic and brand drugs on the primary outcome when a follow-up period was censored on the date of 360, 180, 90, and 30 days from t_0_ (additional analysis). *ATOR: atorvastatin, SIMV: simvastatin, PITA: pitavastatin, PRAV: pravastatin, FLUV: fluvastatin, ROSV: rosuvastatin, FU360, FU180, FU90 and FU30: number of follow-up period (days) censored from t_0_, respectively. aHR: adjusted hazard ratio, NA: not available. † The estimation accuracy of the adjusted hazard ratio was low for the group due to the small number of patients

When the follow-up period was censored on the date of 30, 90, 180 or 360 days after t_0_, results similar to the primary analysis were observed for aHRs on the primary outcome for the five statin cohorts, with the exception of the atorvastatin cohort (Fig. 3). Interestingly, aHRs for the atorvastatin cohort gradually decreased from 1.74 to 1.33 and approached 1.00 (1.74 [95% CI: 0.94—3.22] within 360 days, 1.65 [95% CI: 0.84—3.25] within 180 days, 1.49 [95% CI: 0.73—3.01] within 90 days, and 1.33 [95% CI: 0.60—2.96] within 30 days) as the follow-up period was shortened.

## Discussion

Results from this study suggest that the risks of liver dysfunction by generic statins are generally comparable to those of the brand drugs, although careful consideration of the findings for the simvastatin and fluvastatin cohorts is needed due to their small sample sizes, which may have limited the ability to adequately adjust for confounding factors.

In the atorvastatin cohort, increased risk of liver dysfunction by the generic drug was observed in the primary analysis but was inconsistent with results in the additional analysis, showing a declining trend of aHR with shorter follow-up periods and their 95% CIs with inclusion of 1.00. This additional analysis was conducted based on the report showing that DILI caused by statins usually occurred during a period of 5 days to 3 months [36]. However, the event onset in the generic atorvastatin group was later than that in the brand group, and no significant increase in aHR was observed in the additional analysis with a shorter follow-up period (i.e., 30—90 days after t_0_). These results could not be affected by detection bias because the warning in an atorvastatin labeling, which calls for periodical monitoring of liver function after the statin administration [19], could motivate the medical staff to frequently conduct liver function tests in clinical practice. In fact, median frequency of laboratory tests for several liver functions (i.e., AST and ALT or T-Bill, ALP, and GGT) in each cohort was more than 1.0/100 person-days, with no differences between the generic and brand groups (data not shown). Therefore, the observed increased risk by generic atorvastatin in the primary analysis may have been affected by time-dependent factors, such as a late-onset outcome unrelated to atorvastatin, and does not necessarily indicate a causal relationship with atorvastatin.

One of the key considerations in this study is the potential for exposure misclassification, given that the classification was based on use of prescription data. The actual drug taken by a patient may have differed from what was prescribed (e.g., a generic drug might have been dispensed at the pharmacy even when a brand drug was indicated). Nevertheless, the impact of such misclassification is likely minimal. In the rosuvastatin cohort, the results based on comparison with a historical brand drug were consistent with those derived from a concurrent brand drug. This suggests that exposure misclassification did not introduce critical bias into the analysis. Notably, historical comparison was feasible only for rosuvastatin, as generic versions of other statins were already available in Japan prior to the study’s inclusion period. However, prescribing and dispensing practices across statins within a hospital setting are generally comparable. Moreover, rosuvastatin and other statins share the same approved indication (i.e., dyslipidemia) and are used in similar clinical contexts. Therefore, given that the analysis was conducted using a single data source (i.e., MID-NET^®^), it would be reasonable to assume that the data characteristics across different statins are generally comparable. Accordingly, the findings from the historical comparison involving rosuvastatin may be extrapolated to other statins. While the exact extent of exposure misclassification remains uncertain, it is unlikely in the study to have introduced substantial bias, and the results can thus be interpreted appropriately.

Strengths of this study were the utilization of RWD from MID-NET^®^, a reliable and valuable medical information database in Japan [21, 22]. By using MID-NET^®^, approximately 60–70% of patients in this study were an elderly population aged over 65 years (Table 1), which is the main population of statin users [37]. Liver dysfunction was also evaluated objectively based on laboratory test results from MID-NET^®^. In addition, we used the HDPS method to select many confounders and to precisely estimate aHRs by taking into consideration unclear contributing factors[38, 39] although the effects of residual confounding factors (e.g., alcohol consumption) could not be completely ruled out. As a limitation, the potential effects of exposure misclassification should be carefully considered when interpreting the study results, as discussed above. The generalizability of these results may also be limited because MID-NET^®^ does not include clinics, which are commonly visited by patients taking statins in Japan. Liver dysfunction may be more readily identified in hospitals where regular laboratory testing is conducted, which may potentially lead to an overestimation of events. However, since the analysis was based on relative risk through comparison with a control group (brand drug) and focused on the difference in the risk between generic and brand drugs, no inclusion of clinics is unlikely to have a significant impact on the overall conclusion.

## Conclusion

The results of this database study suggest that the risk of liver dysfunction by generic statins is comparable to that by their corresponding brand drugs, including the atorvastatin cohort. These findings enhance our understanding about the safety of generic drugs, support the use of generic statins in clinical practice, and may partly address concerns regarding benefits/risks of generic drugs in Japan.

## Supplementary Information

Below is the link to the electronic supplementary material.

Supplementary Material 1

Supplementary Material 2

Supplementary Material 3

Supplementary Material 4

Supplementary Material 5

Supplementary Material 6

Supplementary Material 7

## Data Availability

The dataset generated in the study is not publicly available due to the terms of use for MID-NET^®^ to which we adhered when conducting this study; the accessibility of the dataset used for this analysis is restricted to specific authors including the corresponding author in a predetermined secure environment. No outside researchers are allowed to access the dataset.
