# Supplementary material for: Safety Comparison of Risk of Liver Dysfunction between Generic and Brand Statin Drugs Marketed in Japan: A Cohort Study Using MID-NET®
Source: Ther Innov Regul Sci. 2025 Dec 27;60(2):336–45. doi: 10.1007/s43441-025-00904-w (PMC12945947; doi:10.1007/s43441-025-00904-w)
Supplement: Supplementary file 1 — Supplementary Material 1 [file 43441_2025_904_MOESM1_ESM.pdf]

**Title:**

Safety comparison of risk of liver dysfunction between generic and brand statin drugs marketed in Japan: a cohort study using MID-NET®

**Journal name:**

Therapeutic Innovation and Regulatory Sciences

**Authors:**

Hotaka Maruyama, Yuki Kinoshita, Takashi Ando, Jun Okui, Maki Komamine, Kazuhiro Kajiyama, Naoya Horiuchi, and Yoshiaki Uyama*

*** Correspondence:**

Yoshiaki Uyama
uyama-yoshiaki@pmda.go.jp
Center for Regulatory Science,
Pharmaceuticals and Medical Devices Agency,
Kasumigaseki 3-3-2, Chiyoda-ku, Tokyo 100-0013, Japan

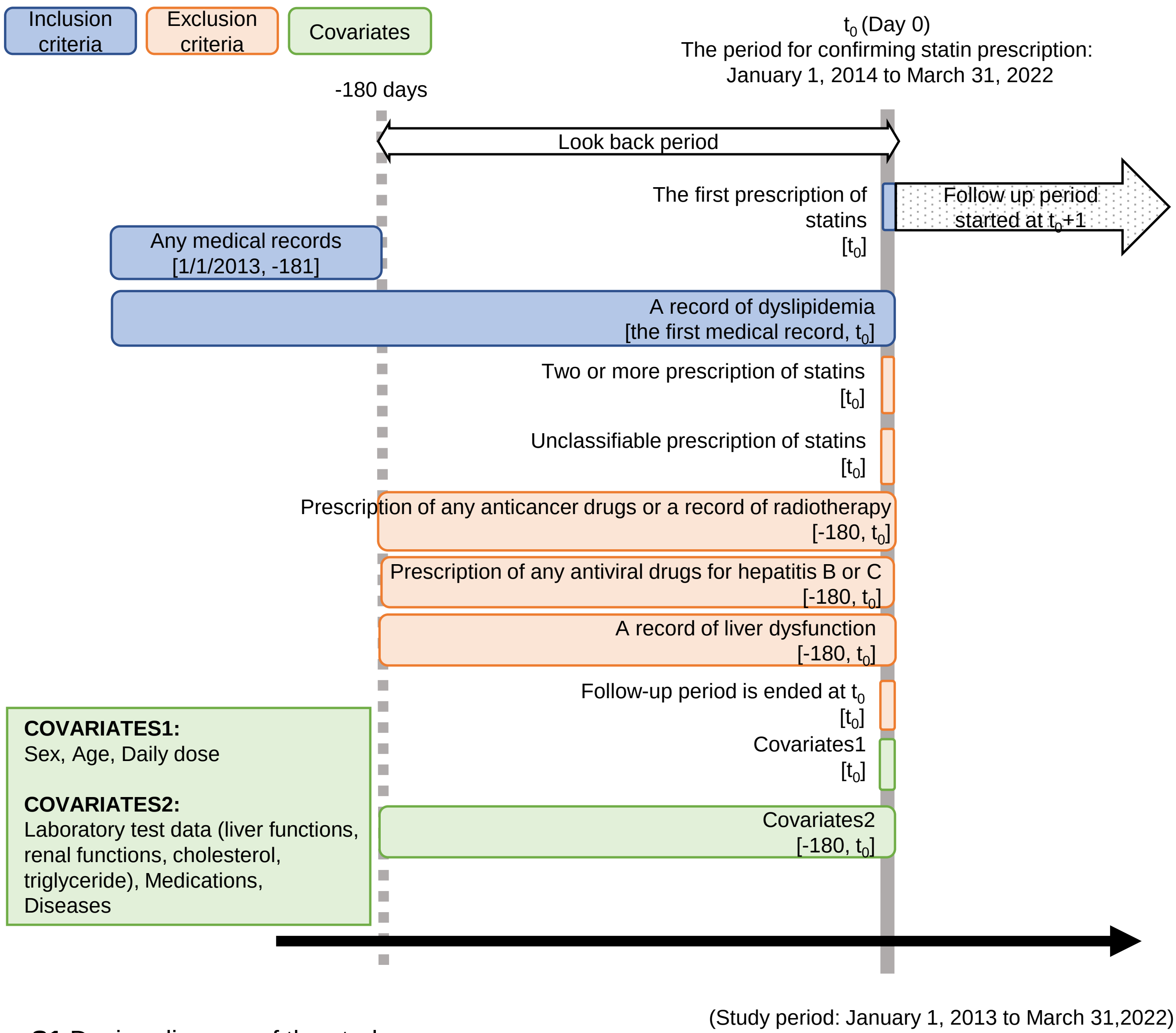


**Fig. S1** Design diagram of the study
