# Supplementary material for: Safety Comparison of Risk of Liver Dysfunction between Generic and Brand Statin Drugs Marketed in Japan: A Cohort Study Using MID-NET®
Source: Ther Innov Regul Sci. 2025 Dec 27;60(2):336–45. doi: 10.1007/s43441-025-00904-w (PMC12945947; doi:10.1007/s43441-025-00904-w)
Supplement: Supplementary file 2 — Supplementary Material 2 [file 43441_2025_904_MOESM2_ESM.pdf]

**Title:**

# Safety comparison of risk of liver dysfunction between generic and brand statin drugs marketed in Japan: a cohort study using MID-NET®

**Journal name:**

Therapeutic Innovation and Regulatory Sciences

**Authors:**

Hotaka Maruyama, Yuki Kinoshita, Takashi Ando, Jun Okui, Maki Komamine, Kazuhiro Kajiyama, Naoya Horiuchi, and Yoshiaki Uyama*

*** Correspondence:**

Yoshiaki Uyama
uyama-yoshiaki@pmda.go.jp
Center for Regulatory Science,
Pharmaceuticals and Medical Devices Agency,
Kasumigaseki 3-3-2, Chiyoda-ku, Tokyo 100-0013, Japan

| Cohort* | Exposure | Number of events‡ | Total follow-up period (person-year) | cHR† [95%CI] | aHR† [95%CI] |
|---|---|---|---|---|---|
| ATOR | Brand | 21 | 3,008.9 | Ref | Ref |
| | Generic | 204 | 14,348.8 | 2.08 [1.33 - 3.26] | 2.54 [1.35 - 4.75] |
| SIMV § | Brand | <10 | 388.1 | Ref | Ref |
| | Generic | <10 | 72.4 | 0.29 [0.04 - 2.33] | 0.13 [0.01 - 1.16] |
| PITV | Brand | 75 | 8,414.5 | Ref | Ref |
| | Generic | 75 | 4,923.8 | 1.54 [1.11 - 2.13] | 1.03 [0.61 - 1.75] |
| PRAV | Brand | 24 | 2,503.7 | Ref | Ref |
| | Generic | 53 | 3,786.8 | 1.39 [0.86 - 2.24] | 0.32 [0.09 - 1.14] |
| FLUV § | Brand | <10 | 425.2 | Ref | Ref |
| | Generic | 0 | 60.0 | NA | NA |
| ROSV | Brand | 148 | 13,161.8 | Ref | Ref |
| | Generic | 20 | 1,501.7 | 1.03 [0.64 - 1.64] | 1.10 [0.61 - 1.97] |

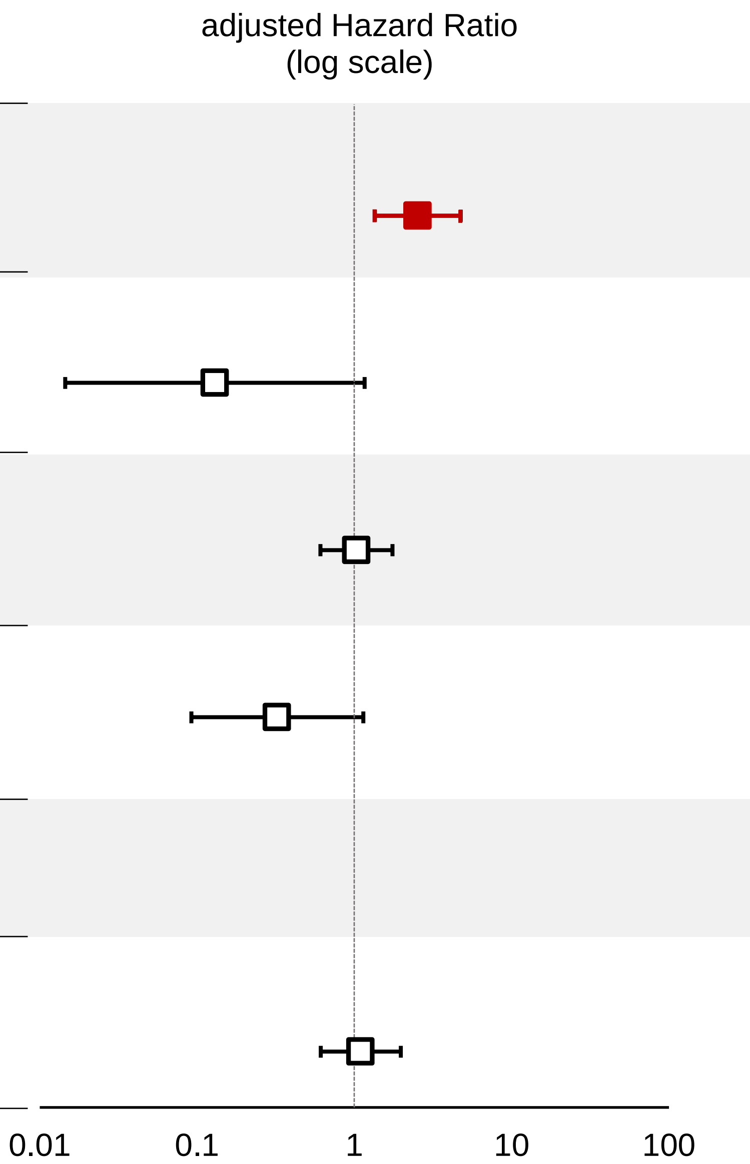


**Fig.** S2 Risk of liver dysfunction by various statins on the secondary outcome (≥ CTCAE grade3)

*ATOR: atorvastatin, SIMV: simvastatin, PITA: pitavastatin, PRAV: pravastatin, FLUV: fluvastatin, ROSV: rosuvastatin

† cHR: crude hazard ratio, aHR: adjusted hazard ratio, Ref: reference

‡ Data are masked so that the number of patients (less than 10) cannot be identified according to the MID-NET® publication criteria.

§ The estimation accuracy of the adjusted hazard ratio was low for groups with a small number of patients
