# Supplementary material for: Safety Comparison of Risk of Liver Dysfunction between Generic and Brand Statin Drugs Marketed in Japan: A Cohort Study Using MID-NET®
Source: Ther Innov Regul Sci. 2025 Dec 27;60(2):336–45. doi: 10.1007/s43441-025-00904-w (PMC12945947; doi:10.1007/s43441-025-00904-w)
Supplement: Supplementary file 3 — Supplementary Material 3 [file 43441_2025_904_MOESM3_ESM.pdf]

Title:

Safety comparison of risk of liver dysfunction between generic and brand statin drugs marketed in Japan: a cohort study using MID-NET®

Journal name:

Therapeutic Innovation and Regulatory Sciences

Authors:

Hotaka Maruyama, Yuki Kinoshita, Takashi Ando, Jun Okui, Maki Komamine, Kazuhiro Kajiyama, Naoya Horiuchi, and Yoshiaki Uyama*

*** Correspondence:**

Yoshiaki Uyama
uyama-yoshiaki@pmda.go.jp
Center for Regulatory Science,
Pharmaceuticals and Medical Devices Agency,
Kasumigaseki 3-3-2, Chiyoda-ku, Tokyo 100-0013, Japan

Supplementary Table S1.1 Characteristics of patients prescribed simvastatin (primary analysis)

Variables*, n (%)		Unadjusted			Adjusted		
		Generic§ (n=261)	Brand (n=363)	ASD†	Generic§ (n=260)	Brand§ (n=257)	ASD†
Sex							
Male		117 (44.8)	149 (41.0)	0.076	117 (45.0)	112 (43.8)	0.025
Age group (years)							
≧65		230 (88.1)	243 (66.9)	0.525	229 (88.1)	227 (88.2)	0.003
Laboratory test result category							
Liver functions1†	Grade1	55 (21.1)	69 (19.0)	0.052	54 (20.8)	57 (22.3)	0.037
Liver functions2†	Grade1	49 (18.8)	88 (24.2)	0.133	49 (18.8)	55 (21.6)	0.066
eGFR†	< 60 mL/min/1.73m^2	118 (45.2)	148 (40.8)	0.090	118 (45.4)	118 (46.1)	0.014
Creatinine Kinase	≧ULN†,‡	28 (10.7)	41 (11.3)	0.018	28 (10.8)	29 (11.3)	0.015
Low Density Lipoprotein	≧140 mg/dL	21 (8.0)	22 (6.1)	0.078	21 (8.1)	19 (7.6)	0.019
High Density Lipoprotein	< 40 mg/dL	<10 (§)	≧60 (§)	0.614	<10 (§)	<10 (§)	0.006
Triglyceride	≧150 mg/dL	54 (20.7)	108 (29.8)	0.210	54 (20.8)	53 (20.6)	0.005
Medications for dyslipidemia							
Other than statins	Yes	17 (6.5)	35 (9.6)	0.115	17 (6.5)	16 (6.1)	0.014
Comorbidities							
Hypertension	Yes	177 (67.8)	207 (57.0)	0.224	177 (68.1)	178 (69.3)	0.025
Diabetes	Yes	170 (65.1)	227 (62.5)	0.054	170 (65.4)	167 (65.0)	0.009
ASO†	Yes	35 (13.4)	46 (12.7)	0.022	35 (13.5)	33 (12.9)	0.016
CAD†	Yes	61 (23.4)	54 (14.9)	0.217	60 (23.1)	56 (21.7)	0.035
CVD†	Yes	88 (33.7)	82 (22.6)	0.249	87 (33.5)	86 (33.5)	0.000
Renal disease	Yes	46 (17.6)	86 (23.7)	0.150	46 (17.7)	45 (17.6)	0.001
Fatty liver disease	Yes	<10 (§)	≧10 (§)	0.102	<10 (§)	<10 (§)	0.017
Other liver disease	Yes	28 (10.7)	56 (15.4)	0.140	28 (10.8)	29 (11.3)	0.015

*This table presents basic covariates other than covariates selected through the method of hdPS.

† ASD, absolute standardized means difference; ASO, arteriosclerosis obliterans, CAD, coronary artery disease; CVD, cerebral vascular disease; eGFR, estimated glomerular filtration rate; Liver functions 1, aspartate aminotransferase (AST) or alanine aminotransferase (ALT); Liver functions 2, gamma glutamyl transferase (GGT), total-bilirubin (T-Bil) or alkaline phosphatase (ALP); ULN, upper limit normal

‡ ULN, 248 U/L (Male), 153 U/L (Female)

§ Data are masked so that the number of patients (less than 10) cannot be identified according to the MID-NET® publication criteria.
