# Supplementary material for: Safety Comparison of Risk of Liver Dysfunction between Generic and Brand Statin Drugs Marketed in Japan: A Cohort Study Using MID-NET®
Source: Ther Innov Regul Sci. 2025 Dec 27;60(2):336–45. doi: 10.1007/s43441-025-00904-w (PMC12945947; doi:10.1007/s43441-025-00904-w)
Supplement: Supplementary file 4 — Supplementary Material 4 [file 43441_2025_904_MOESM4_ESM.pdf]

Title:

Safety comparison of risk of liver dysfunction between generic and brand statin drugs marketed in Japan: a cohort study using MID-NET®

Journal name:

Therapeutic Innovation and Regulatory Sciences

Authors:

Hotaka Maruyama, Yuki Kinoshita, Takashi Ando, Jun Okui, Maki Komamine, Kazuhiro Kajiyama, Naoya Horiuchi, and Yoshiaki Uyama*

*** Correspondence:**

Yoshiaki Uyama

uyama-yoshiaki@pmda.go.jp

Center for Regulatory Science,

Pharmaceuticals and Medical Devices Agency,

Kasumigaseki 3-3-2, Chiyoda-ku, Tokyo 100-0013, Japan

Supplementary Table S1.2 Characteristics of patients prescribed pitavastatin (primary analysis)

Variables*, n (%)		Unadjusted			Adjusted		
		Generic (n=4,939)	Brand (n=6,493)	ASD†	Generic (n=4,913)	Brand (n=5,293)	ASD†
Sex							
Male		2,710 (54.9)	3,552 (54.7)	0.003	2,696 (54.9)	2,837 (53.6)	0.026
Age group (years)							
≧65		3,261 (66.0)	3,968 (61.1)	0.102	3,240 (65.9)	3,610 (68.2)	0.047
Laboratory test result category							
Liver functions1†	Grade1	1,107 (22.4)	1,400 (21.6)	0.021	1,098 (22.3)	1,179 (22.3)	0.002
Liver functions2†	Grade1	1,154 (23.4)	1,484 (22.9)	0.012	1,147 (23.3)	1,266 (23.9)	0.014
eGFR†	< 60 mL/min/1.73m²	1,999 (40.5)	2,430 (37.4)	0.063	1,983 (40.4)	2,255 (42.6)	0.046
Creatinine Kinase	≧ULN†,‡	534 (10.8)	646 (9.9)	0.028	525 (10.7)	585 (11.1)	0.012
Low Density Lipoprotein	≧140 mg/dL	595 (12.0)	731 (11.3)	0.025	589 (12.0)	609 (11.5)	0.015
High Density Lipoprotein	< 40 mg/dL	941 (19.1)	1,665 (25.6)	0.159	938 (19.1)	935 (17.7)	0.035
Triglyceride	≧150 mg/dL	1,590 (32.2)	2,171 (33.4)	0.026	1,583 (32.2)	1,851 (35.0)	0.058
Medications for dyslipidemia							
Other than statins	Yes	612 (12.4)	695 (10.7)	0.053	608 (12.4)	760 (14.4)	0.062
Comorbidities							
Hypertension	Yes	3,097 (62.7)	4,065 (62.6)	0.002	3,077 (62.6)	3,217 (60.8)	0.038
Diabetes	Yes	3,557 (72.0)	4,712 (72.6)	0.012	3,534 (71.9)	3,896 (73.6)	0.037
ASO†	Yes	967 (19.6)	1,540 (23.7)	0.101	960 (19.5)	1,076 (20.3)	0.019
CAD†	Yes	1,337 (27.1)	1,770 (27.3)	0.004	1,328 (27.0)	1,458 (27.6)	0.012
CVD†	Yes	1,988 (40.3)	2,584 (39.8)	0.009	1,976 (40.2)	2,154 (40.7)	0.009
Renal disease	Yes	890 (18.0)	1,059 (16.3)	0.045	876 (17.8)	1,074 (20.3)	0.066
Fatty liver disease	Yes	217 (4.4)	380 (5.9)	0.066	217 (4.4)	243 (4.6)	0.008
Other liver disease	Yes	744 (15.1)	1,154 (17.8)	0.073	742 (15.1)	905 (17.1)	0.054

*This table presents basic covariates other than covariates selected through the method of hdPS.

† ASD, absolute standardized means difference; ASO, arteriosclerosis obliterans; CAD, coronary artery disease; CVD, cerebral vascular disease; eGFR, estimated glomerular filtration rate; Liver functions 1, aspartate aminotransferase (AST) or alanine aminotransferase (ALT); Liver functions 2, gamma glutamyl transferase (GGT), total-bilirubin (T-Bil) or alkaline phosphatase (ALP); ULN, upper limit normal

‡ ULN, 248 U/L (Male), 153 U/L (Female)
