# Supplementary material for: Safety Comparison of Risk of Liver Dysfunction between Generic and Brand Statin Drugs Marketed in Japan: A Cohort Study Using MID-NET®
Source: Ther Innov Regul Sci. 2025 Dec 27;60(2):336–45. doi: 10.1007/s43441-025-00904-w (PMC12945947; doi:10.1007/s43441-025-00904-w)
Supplement: Supplementary file 5 — Supplementary Material 5 [file 43441_2025_904_MOESM5_ESM.pdf]

Title:

Safety comparison of risk of liver dysfunction between generic and brand statin drugs marketed in Japan: a cohort study using MID-NET®

Journal name:

Therapeutic Innovation and Regulatory Sciences

Authors:

Hotaka Maruyama, Yuki Kinoshita, Takashi Ando, Jun Okui, Maki Komamine, Kazuhiro Kajiyama, Naoya Horiuchi, and Yoshiaki Uyama*

*** Correspondence:**

Yoshiaki Uyama
uyama-yoshiaki@pmda.go.jp
Center for Regulatory Science,
Pharmaceuticals and Medical Devices Agency,
Kasumigaseki 3-3-2, Chiyoda-ku, Tokyo 100-0013, Japan

Supplementary Table S1.3 Characteristics of patients prescribed pravastatin (primary analysis)

Variables*, n (%)		Unadjusted			Adjusted		
		Generic (n=3,893)	Brand (n=2,267)	ASD[†]	Generic (n=3,774)	Brand (n=4,333)	ASD[†]
Sex							
Male		1,695 (43.5)	979 (43.2)	0.007	1,649 (43.7)	1,682 (38.8)	0.098
Age group (years)							
≧65		2,894 (74.3)	1,539 (67.9)	0.143	2,791 (74.0)	3,398 (78.4)	0.098
Laboratory test result category							
Liver functions1[†]	Grade1	817 (21.0)	500 (22.1)	0.026	794 (21.0)	819 (18.9)	0.052
Liver functions2[†]	Grade1	921 (23.7)	549 (24.2)	0.013	888 (23.5)	954 (22.0)	0.036
eGFR[†]	< 60 mL/min/1.73m^2	1,535 (39.4)	878 (38.7)	0.014	1,478 (39.2)	1,841 (42.5)	0.068
Creatinine Kinase	≧ULN[†,‡]	429 (11.0)	212 (9.4)	0.055	411 (10.9)	383 (8.8)	0.068
Low Density Lipoprotein	≧140 mg/dL	439 (11.3)	180 (7.9)	0.113	403 (10.7)	534 (12.3)	0.057
High Density Lipoprotein	< 40 mg/dL	701 (18.0)	434 (19.1)	0.029	679 (18.0)	445 (10.3)	0.198
Triglyceride	≧150 mg/dL	1,054 (27.1)	626 (27.6)	0.012	1,014 (26.9)	1,158 (26.7)	0.003
Medications for dyslipidemia							
Other than statins	Yes	313 (8.0)	215 (9.5)	0.051	305 (8.1)	377 (8.7)	0.022
Comorbidities							
Hypertension	Yes	2,512 (64.5)	1,306 (57.6)	0.142	2,421 (64.1)	2,899 (66.9)	0.057
Diabetes	Yes	2,355 (60.5)	1,581 (69.7)	0.195	2,285 (60.5)	2,510 (57.9)	0.055
ASO[†]	Yes	540 (13.9)	354 (15.6)	0.049	525 (13.9)	577 (13.3)	0.017
CAD[†]	Yes	1,072 (27.5)	500 (22.1)	0.127	1,039 (27.5)	1,240 (28.6)	0.025
CVD[†]	Yes	1,103 (28.3)	634 (28.0)	0.008	1,052 (27.9)	1,318 (30.4)	0.057
Renal disease	Yes	511 (13.1)	439 (19.4)	0.170	491 (13.0)	597 (13.8)	0.021
Fatty liver disease	Yes	164 (4.2)	94 (4.1)	0.003	162 (4.3)	220 (5.1)	0.039
Other liver disease	Yes	572 (14.7)	414 (18.3)	0.096	556 (14.7)	640 (14.8)	0.001

*This table presents basic covariates other than covariates selected through the method of hdPS.

[†] ASD, absolute standardized means difference; ASO, arteriosclerosis obliterans; CAD, coronary artery disease; CVD, cerebral vascular disease; eGFR, estimated glomerular filtration rate; Liver functions 1, aspartate aminotransferase (AST) or alanine aminotransferase (ALT); Liver functions 2, gamma glutamyl transferase (GGT), total-bilirubin (T-Bil) or alkaline phosphatase (ALP); ULN, upper limit normal

[‡] ULN, 248 U/L (Male), 153 U/L (Female)
