# Supplementary material for: Safety Comparison of Risk of Liver Dysfunction between Generic and Brand Statin Drugs Marketed in Japan: A Cohort Study Using MID-NET®
Source: Ther Innov Regul Sci. 2025 Dec 27;60(2):336–45. doi: 10.1007/s43441-025-00904-w (PMC12945947; doi:10.1007/s43441-025-00904-w)
Supplement: Supplementary file 6 — Supplementary Material 6 [file 43441_2025_904_MOESM6_ESM.pdf]

Title:

Safety comparison of risk of liver dysfunction between generic and brand statin drugs marketed in Japan: a cohort study using MID-NET®

Journal name:

Therapeutic Innovation and Regulatory Sciences

Authors:

Hotaka Maruyama, Yuki Kinoshita, Takashi Ando, Jun Okui, Maki Komamine, Kazuhiro Kajiyama, Naoya Horiuchi, and Yoshiaki Uyama*

*** Correspondence:**

Yoshiaki Uyama
uyama-yoshiaki@pmda.go.jp
Center for Regulatory Science,
Pharmaceuticals and Medical Devices Agency,
Kasumigaseki 3-3-2, Chiyoda-ku, Tokyo 100-0013, Japan

Supplementary Table S1.4 Characteristics of patients prescribed fluvastatin (primary analysis

Variables*, n (%)		Unadjusted			Adjusted		
		Generic[§] (n=106)	Brand[§] (n=325)	ASD[†]	Generic[§] (n=102)	Brand[§] (n=105)	ASD[†]
Sex							
Male		43 (40.6)	149 (45.8)	0.107	43 (42.2)	42 (40.4)	0.036
Age group (years)							
≧65		79 (74.5)	192 (59.1)	0.333	75 (73.5)	80 (75.8)	0.048
Laboratory test result category							
Liver functions1[†]	Grade1	23 (21.7)	58 (17.8)	0.097	20 (19.6)	21 (20.1)	0.013
Liver functions2[†]	Grade1	30 (28.3)	65 (20.0)	0.195	26 (25.5)	29 (27.8)	0.054
eGFR[†]	< 60 mL/min/1.73m^2	47 (44.3)	137 (42.2)	0.044	45 (44.1)	48 (45.4)	0.027
Creatinine Kinase	≧ULN[†,‡]	<10 ([§])	≧10 ([§])	0.004	<10 ([§])	<10 ([§])	0.004
Low Density Lipoprotein	≧140 mg/dL	<10 ([§])	≧10 ([§])	0.067	<10 ([§])	≧10 ([§])	0.011
High Density Lipoprotein	< 40 mg/dL	15 (14.2)	81 (24.9)	0.274	15 (14.7)	15 (14.2)	0.014
Triglyceride	≧150 mg/dL	27 (25.5)	86 (26.5)	0.023	25 (24.5)	27 (26.0)	0.034
Medications for dyslipidemia							
Other than statins	Yes	<10 ([§])	≧30 ([§])	0.097	<10 ([§])	<10 ([§])	0.004
Comorbidities							
Hypertension	Yes	67 (63.2)	220 (67.7)	0.094	64 (62.7)	67 (63.5)	0.015
Diabetes	Yes	78 (73.6)	223 (68.6)	0.110	74 (72.5)	77 (73.6)	0.024
ASO[†]	Yes	16 (15.1)	68 (20.9)	0.152	16 (15.7)	16 (14.9)	0.021
CAD[†]	Yes	26 (24.5)	67 (20.6)	0.094	25 (24.5)	26 (25.2)	0.016
CVD[†]	Yes	40 (37.7)	122 (37.5)	0.004	39 (38.2)	39 (37.4)	0.017
Renal disease	Yes	26 (24.5)	75 (23.1)	0.034	25 (24.5)	28 (26.4)	0.045
Fatty liver disease	Yes	<10 ([§])	≧10 ([§])	0.238	<10 ([§])	<10 ([§])	0.032
Other liver disease	Yes	14 (13.2)	67 (20.6)	0.199	14 (13.7)	14 (13.4)	0.009

*This table presents basic covariates other than covariates selected through the method of hdPS.

[†]ASD, absolute standardized means difference; ASO, arteriosclerosis obliterans, CAD, coronary artery disease; CVD, cerebral vascular disease; eGFR, estimated glomerular filtration rate; Liver functions 1, aspartate aminotransferase (AST) or alanine aminotransferase (ALT); Liver functions 2, gamma glutamyl transferase (GGT), total-bilirubin (T-Bil) or alkaline phosphatase (ALP); ULN, upper limit normal

[‡]ULN, 248 U/L (Male), 153 U/L (Female)

[§]Data are masked so that the number of patients (less than 10) cannot be identified according to the MID-NET® publication criteria.
