# Supplementary material for: Safety Comparison of Risk of Liver Dysfunction between Generic and Brand Statin Drugs Marketed in Japan: A Cohort Study Using MID-NET®
Source: Ther Innov Regul Sci. 2025 Dec 27;60(2):336–45. doi: 10.1007/s43441-025-00904-w (PMC12945947; doi:10.1007/s43441-025-00904-w)
Supplement: Supplementary file 7 — Supplementary Material 7 [file 43441_2025_904_MOESM7_ESM.pdf]

Title:

Safety comparison of risk of liver dysfunction between generic and brand statin drugs marketed in Japan: a cohort study using MID-NET®

Journal name:

Therapeutic Innovation and Regulatory Sciences

Authors:

Hotaka Maruyama, Yuki Kinoshita, Takashi Ando, Jun Okui, Maki Komamine, Kazuhiro Kajiyama, Naoya Horiuchi, and Yoshiaki Uyama*

*** Correspondence:**

Yoshiaki Uyama
uyama-yoshiaki@pmda.go.jp
Center for Regulatory Science,
Pharmaceuticals and Medical Devices Agency,
Kasumigaseki 3-3-2, Chiyoda-ku, Tokyo 100-0013, Japan

Supplementary Table S1.5 Characteristics of patients prescribed rosuvastatin (primary analysis)

Variables*, n (%)		Unadjusted			Adjusted		
		Generic (n=2,239)	Brand (n=13,715)	ASD†	Generic (n=2,214)	Brand (n=2,217)	ASD†
Sex							
Male		1,180 (52.7)	7,529 (54.9)	0.044	1,168 (52.8)	1,125 (50.7)	0.041
Age group (years)							
≧65		1,387 (61.9)	8,473 (61.8)	0.003	1,367 (61.7)	1,392 (62.8)	0.021
Laboratory test result category							
Liver functions1†	Grade1	545 (24.3)	3,160 (23.0)	0.031	540 (24.4)	528 (23.8)	0.013
Liver functions2†	Grade1	542 (24.2)	3,190 (23.3)	0.022	533 (24.1)	534 (24.1)	0.000
eGFR†	< 60 mL/min/1.73m^2	864 (38.6)	5,254 (38.3)	0.006	854 (38.6)	840 (37.9)	0.014
Creatinine Kinase	≧ULN†,‡	239 (10.7)	1,430 (10.4)	0.008	235 (10.6)	231 (10.4)	0.006
Low Density Lipoprotein	≧140 mg/dL	230 (10.3)	1,544 (11.3)	0.032	228 (10.3)	221 (10.0)	0.011
High Density Lipoprotein	< 40 mg/dL	567 (25.3)	4,194 (30.6)	0.117	561 (25.3)	511 (23.1)	0.051
Triglyceride	≧150 mg/dL	746 (33.3)	4,842 (35.3)	0.042	738 (33.3)	692 (31.2)	0.045
Medications for dyslipidemia							
Other than statins	Yes	215 (9.6)	1,324 (9.7)	0.002	214 (9.7)	217 (9.8)	0.004
Comorbidities							
Hypertension	Yes	1,353 (60.4)	8,211 (59.9)	0.011	1,334 (60.3)	1,311 (59.1)	0.023
Diabetes	Yes	1,550 (69.2)	9,493 (69.2)	0.000	1,529 (69.1)	1,502 (67.7)	0.029
ASO†	Yes	346 (15.5)	2,257 (16.5)	0.027	338 (15.3)	305 (13.7)	0.042
CAD†	Yes	427 (19.1)	3,878 (28.3)	0.218	423 (19.1)	411 (18.5)	0.013
CVD†	Yes	813 (36.3)	5,079 (37.0)	0.015	797 (36.0)	757 (34.2)	0.038
Renal disease	Yes	283 (12.6)	1,932 (14.1)	0.043	281 (12.7)	278 (12.5)	0.005
Fatty liver disease	Yes	91 (4.1)	758 (5.5)	0.068	91 (4.1)	92 (4.2)	0.002
Other liver disease	Yes	291 (13.0)	2,215 (16.2)	0.089	288 (13.0)	284 (12.8)	0.006

*This table presents basic covariates other than covariates selected through the method of hdPS.

†ASD, absolute standardized means difference; ASO, arteriosclerosis obliterans; CAD, coronary artery disease; CVD, cerebral vascular disease; eGFR, estimated glomerular filtration rate; Liver functions 1, aspartate aminotransferase (AST) or alanine aminotransferase (ALT); Liver functions 2, gamma glutamyl transferase (GGT), total-bilirubin (T-Bil) or alkaline phosphatase (ALP); ULN, upper limit normal

‡ULN, 248 U/L (Male), 153 U/L (Female)
